# Supplementary material for: Trehalose metabolism and its impact on PrfA activity in Listeria monocytogenes
Source: J Bacteriol. 2026 Jul 14;208(8):e00247-26. doi: 10.1128/jb.00247-26 (PMC13492111; doi:10.1128/jb.00247-26)
Supplement: Supplementary file 1 [file jb.00247-26-s0001.docx]

**Supplemental Material**

**Trehalose metabolism and its impact on PrfA activity in *Listeria monocytogenes***

Jessica Schüler^a^, Annette Walz^b*^, Niclas Wüstefeld^a^, Maja Andiel^a^, Wolfgang Eisenreich^b^, Jeanine Rismondo^a#^

^a^ Department of General Microbiology, Institute of Microbiology and Genetics, GZMB, Georg-August University Göttingen, Göttingen, Germany

^b^ Structural Membrane Biochemistry, BNMRZ, Department of Chemistry, Technische Universität München, Garching, Germany

Running title: Impact of trehalose on PrfA activity

\# Corresponding author, jrismon@gwdg.de

* Present address: Department of Bioinformatics and Biochemistry, Braunschweig Integrated Centre of Systems Biology (BRICS), Technische Universität Braunschweig, Braunschweig, Germany

**Supplemental Tables**

**Table S1: Bacterial strains used in this study**

| Unique ID | Strain name and resistance | Source |
| --- | --- | --- |
| *Escherichia coli* strains | | |
| ANG124 | DH5α pKSV7; AmpR | (1) |
| ANG4242 | XL1-Blue pIMK2; KanR | (2) |
| ANG5181 | XL1-Blue pPL3e-*lacZ*; CamR | (3) |
| EJR274 | XL1-Blue pPL3e-*P_plcA_-P_prfA_*-*prfA*; cat^R^ | This study |
| EJR286 | DH5α pPL3e-*P_actA_-lacZ*; CamR | This study |
| EJR378 | DH5α pKSV7-Δ*celR*; AmpR | This study |
| EJR379 | DH5α pKSV7-Δ*manR*; AmpR | This study |
| EJR382 | DH5α pPL3e-*P_treBA_-lacZ*; CamR | This study |
| EJR383 | DH5α pIMK2-*treBA*; KanR | This study |
| EJR385 | DH5α pIMK2-*treB*; KanR | This study |
| EJR388 | DH5α pKSV7-Δ*treA*; AmpR | This study |
| EJR389 | DH5α pKSV7-Δ*treB*; AmpR | This study |
| EJR390 | DH5α pKSV7-Δ*treR*; AmpR | This study |
| EJR403 | S17-1 pPL3e-*P_treBA_-lacZ*; CamR | This study |
| EJR404 | DH5α pKSV7-Δ*lmo1017*; AmpR | This study |
| EJR409 | DH5α pWH844-*treR*; AmpR | This study |
| EJR415 | DH5α pKSV7-Δ*lmo0862*; AmpR | This study |
| EJR417 | DH5α pIMK2-*lmo0862*; KanR | This study |
| EJR443 | DH5α pKSV7-Δ*lmo0184*; AmpR | This study |
| EJR444 | DH5α pIMK2-*lmo0184*; KanR | This study |
| *Listeria monocytogenes* strains | | |
| ANG873 | EGD-e | (4) |
| BUG2214 | EGD-e Δ*prfA* | (5) |
| LJR334 | EGD-e Δ*prfA* pPL3e-*P_plcA_-P_prfA_*-*prfA*; ery^R^; short: Δ*prfA* compl. | This study |
| LJR352 | EGD-e pPL3e-*P_actA_-lacZ*; ErmR | This study |
| LJR436 | EGD-e Δ*celR* | This study |
| LJR584 | EGD-e Δ*manR* | This study |
| LJR622 | EGD-e pPL3e-*P_treBA_-lacZ*; ErmR | This study |
| LJR644 | EGD-e pIMK2-*treB*, KanR | This study |
| LJR646 | EGD-e pIMK2-*treBA*, KanR | This study |
| LJR717 | EGD-e Δ*treA* | This study |
| LJR718 | EGD-e Δ*treB* | This study |
| LJR719 | EGD-e Δ*treR* | This study |
| LJR730 | EGD-e Δ*treR* pPL3e-*P_treBA_-lacZ*; ErmR | This study |
| LJR746 | EGD-e Δ*lmo1017* | This study |
| LJR760 | EGD-e pIMK2-*lmo0862*; KanR | This study |
| LJR761 | EGD-e Δ*treA* pIMK2-*lmo0862*; KanR | This study |
| LJR776 | EGD-e pIMK2-*lmo0184*; KanR | This study |
| LJR778 | EGD-e Δ*treA* pIMK2-*lmo0184*; KanR | This study |
| LJR791 | EGD-e Δ*lmo0862* | This study |
| LJR792 | EGD-e Δ*treA* Δ*lmo0862* | This study |
| LJR793 | EGD-e Δ*treA* Δ*lmo0184* | This study |
| LJR794 | EGD-e Δ*lmo0184* | This study |

**Table S2: Primers used in this study**

| **Number** | **Name** | **Sequence** |
| --- | --- | --- |
| JR257 | *P_plcA_-P_prfA_-prfA* rev | ACGCGTCGACCGAATAAAATATAAAACAGTATTCCTC |
| JR312 | *P_plcA_-P_prfA_-prfA* fw | CGGGGTACCGAAATTCGCTTCTAAAGATGAAACG |
| JR434 | *treB* up rev | AGATTCACGATAAGCGCTTGCATCCTTTTTATAGTC |
| JR435 | *treB* down fw | GATGCAAGCGCTTATCGTGAATCTGCAAAAACAACCAAC |
| JR465 | *manR* up fw | AAAGGTACCCCATCCTAATTCATCCCTTTCG |
| JR466 | *manR* up rev | TTGGATTTCATTAAATTCATAAATACGGTCAATACGCTTC |
| JR467 | *manR* down fw | ATTTATGAATTTAATGAAATCCAACCAGAAGTACTTTC |
| JR468 | *manR* down rev | TTTGTCGACCTTCTACGAGGGTTAGTACATC |
| JR471 | *celR* up fw | AAAGGTACCGCGACAAAACTAGAAAATCCGG |
| JR472 | *celR* up rev | ATTGGAGAGCATTTGGAGGACTTCTTCTTTTCTACTA |
| JR473 | *celR* down fw | GAAGTCCTCCAAATGCTCTCCAATCAGGTAGAAG |
| JR474 | *celR* down rev | TTTGTCGACGCTACATGTTGTGGAAATACGG |
| JR489 | *P_treBA_* fw | AAAGGATCCCATTTTCAACAGCGCCACCC |
| JR490 | *P_treBA_* rev | TTTGTCGACATAGTCAACCATAAAATTTCCTCCTTTTTAAC |
| JR491 | pIMK2-*treBA* fw | AAACCATGGTTGACTATAAAAAGGATGCAAGCG |
| JR492 | pIMK2-*treBA* rev | TTTGTCGACTTATTGTATTAATGTTAATGTTTCATATGGCG |
| JR493 | pIMK2-*treB* rev | TTTGTCGACTTAGTTGGTTGTTTTTGCAGATTCAC |
| JR494 | *treA* up fw | AAAGGTACCGGTGTTTATAGTTTCCTTTGGCTC |
| JR495 | *treA* up rev | TGTTTCATATGGATAAATTGTTTTTTGAGCAAACTTTGTCAT |
| JR496 | *treA* down fw | AAAACAATTTATCCATATGAAACATTAACATTAATACAATAA |
| JR497 | *treA* down rev | TTTGTCGACCTAACAAATTTCGGTTACAATTAGGG |
| JR501 | *treR* up rev | ATCAATAAAACGAATGTCAAAAAACTTATTTTTCTTATTCAAATTT |
| JR502 | *treR* down fw | TTTTTTGACATTCGTTTTATTGATTTTGCTAGAAGACG |
| JR507 | *treB* up fw | AAAGGTACCCCGTTCTGCCCAAACTGTTC |
| JR508 | *treB* down rev | TTTGTCGACGTACATTCGGATTGGCCCAG |
| JR511 | *treR* up fw | AAAGGTACCCCTCTAAATAGGCACTCTGGAG |
| JR512 | *treR* down rev | TTTGTCGACCGGACTTAGTGAGCAGGAAG |
| JR551 | pIMK2-*lmo0862* fw | AAAGGATCCCGAGTTTTGGCGTCGTAGTGTG |
| JR552 | pIMK2-*lmo0862* rev | TTTGTCGACTTATTCCTCCACTCTAAGCGC |
| JR561 | pIMK2-*lmo0184* fw | AAAGGATCCCAAAGAAAAAGATTGGTGGAAAAAAAGC |
| JR562 | pIMK2-*lmo0184* rev | TTTGTCGACTTACTTGATTTGATAAACGATTGCTTC |
| JR563 | *lmo0184* up fw | AAAGGTACCGGGCTTTTGGTGCGGACG |
| JR564 | *lmo0184* up rev | GATTGCTTCGTAGCTTTTTTTCCACCAATCTTTTTCTTTC |
| JR565 | *lmo0184* down fw | TGGAAAAAAAGCTACGAAGCAATCGTTTATCAAATCAAG |
| JR566 | *lmo0184* down rev | TTTGTCGACGCATAATTCCGCCTACTTCTTC |
| JS21 | *lmo0862* up fw | AAAGGATCCCGCTATATACTGACAACATTACTC |
| JS22 | *lmo0862*up rev | GTTCCCCTGAAGAAACACACTACGACGCCAAAAC |
| JS23 | *lmo0862*down fw | CGTAGTGTGTTTCTTCAGGGGAACTATCAGCTT |
| JS24 | *lmo0862*down rev | TTTGTCGACCGATGTGACCAAGCACGC |
| NW11 | *lmo1017* up fw | AAAGGTACCCAATCCAGATAAAGTAGCAAAATGGA |
| NW12 | *lmo1017* up rev | GTTTTCCCTGCTTCCGCCATGCATCTTCCTCCTAGCAAT |
| NW13 | *lmo1017* down fw | CTAGGAGGAAGATGCATGGCGGAAGCAGGGAAAACC |
| NW14 | *lmo1017* down rev | AAAGTCGACGGTAACTCGCTTCGATATCTTTG |
| NW21 | pWH844-*treR* fw | AAAGGATCCTTGAATAAGAAAAATAAGTTTTTTGACATTTATTTAG |
| NW22 | pWH844-*treR* rev | AAAGTCGACTTAGCGTCTTCTAGCAAAATCAATAAAAC |
| NW23 | EMSA *P_treBA_* fw | ATAGTCAACCATAAAATTTCCTCCTTTTTAAC |
| NW24 | EMSA *P_treBA_* rev | CATTTTCAACAGCGCCACCC |
| NW25 | EMSA *P_cadA_* fw | CGTACAAGACATACCGTCTAC |
| NW26 | EMSA *P_cadA_* rev | CATTTACTTACCTTAAGACAAG |

**Supplemental Figures**

**Fig. S1: Enhanced growth of *L. monocytogenes* in the presence of trehalose.** Overnight cultures of the *L. monocytogenes* wildtype strain EGD-e were diluted to an OD_600_ of 0.1 in fresh LB medium. When the cultures reached an OD_600_, cells were collected, washed twice in LB medium or LB medium containing 25 mM trehalose and OD_600_ adjusted as described in the methods section. The growth was monitored for 25 hours using a plate reader. The average values and standard deviations of the OD_600_ readings of three independent experiments were plotted.

**Fig. S2:** **Growth of *treA*, *treB*, *treR* and *lmo1017* deletion strains on LSM glucose and cellobiose.** (A, C) The indicated *L. monocytogenes* strains were grown overnight in BHI broth, diluted to an OD_600_ of 0.1 in fresh BHI broth and grown until an OD_600_ of 0.3 was reached. (B, D) *L. monocytogenes* strains were grown overnight in LSM glucose, diluted to an OD_600_ of 0.1 in fresh LSM glucose and grown until an OD_600_ of 0.3 was reached. (A-D) Cells were collected, washed and OD_600_ adjusted as described in the methods section. The growth was monitored for 25 hours using a plate reader. The average values and standard deviations of the OD_600_ readings of three independent experiments were plotted.

**Fig. S3: Lmo0184 und Lmo0862 are not required for trehalose metabolism.** (A-B) *L. monocytogenes* strains were grown overnight in LSM glucose, diluted to an OD_600_ of 0.1 in fresh LSM glucose and grown until an OD_600_ of 0.3 was reached. (C) The indicated *L. monocytogenes* strains were grown overnight in BHI broth, diluted to an OD_600_ of 0.1 in fresh BHI broth and grown until an OD_600_ of 0.3 was reached. (A-C) Cells were collected, washed and OD_600_ adjusted as described in the methods section. The growth was monitored for 25 hours using a plate reader. The average values and standard deviations of the OD_600_ readings of three independent experiments were plotted.

**Fig. S4: Overexpression of *treB* leads to growth defect in LSM trehalose.** (A) PlcB activity of the *L. monocytogenes* wildtype strain EGD-e (WT), the *treB* overexpression strain (LJR644; WT + *treB*), the *treBA* overexpression strain (LJR646; WT + *treBA*) and the *prfA* mutant was tested on LB agar plates containing activated charcoal and egg yolk or on plates that additionally contained 25 mM trehalose. (B) Overnight cultures of the *L. monocytogenes* wildtype strain EGD-e, the *treB* overexpression strain and the *treBA* overexpression strain were diluted to an OD_600_ of 0.1 in fresh BHI medium. When the cultures reached an OD_600_, cells were collected, washed and OD_600_ adjusted as described in the methods section. The growth in LSM glucose and LSM trehalose was monitored for 25 hours using a plate reader. The average values and standard deviations of the OD_600_ readings of three independent experiments were plotted.

**Fig. S5: TreR binds to the *P_treBA_* promoter region.** Increasing concentrations of recombinant His-TreR were incubated with a 236-bp fragment containing the *treBA* promoter (lanes 4-7). A reaction of 2 µmol TreR with a 200-bp fragment containing the *cadA* promoter was used as negative control (lane 2). Reactions without protein were used as additional control (-).

**Fig. S6: Growth of *manR*, *celR* and *treR* mutants.** *L. monocytogenes* strains were grown overnight in LSM glucose, diluted to an OD_600_ of 0.1 in fresh LSM glucose and grown until an OD_600_ of 0.3 was reached. Cells were collected, washed and OD_600_ adjusted as described in the methods section. The growth was monitored for 25 hours using a plate reader. The average values and standard deviations of the OD_600_ readings of three independent experiments were plotted.

**References**

1.	Smith K, Youngman P. 1992. Use of a new integrational vector to investigate compartment-specific expression of the *Bacillus subtilis spollM* gene. Biochimie 74:705–711.

2.	Monk IR, Gahan CGM, Hill C. 2008. Tools for functional postgenomic analysis of *Listeria monocytogenes*. Appl Environ Microbiol 74:3921–3934.

3.	Rismondo J, Halbedel S, Gründling A. 2019. Cell shape and antibiotic resistance are maintained by the activity of multiple FtsW and RodA enzymes in *Listeria monocytogenes*. mBio 10:e01448-19.

4.	Glaser P, Frangeul L, Buchrieser C, Rusniok C, Amend A, Baquero F, Berche P, Bloecker H, Brandt P, Chakraborty T, Charbit A, Chetouani F, Couvé E, de Daruvar A, Dehoux P, Domann E, Domı́nguez-Bernal G, Duchaud E, Durant L, Dussurget O, Entian K-D, Fsihi H, Portillo FG-D, Garrido P, Gautier L, Goebel W, Gómez-López N, Hain T, Hauf J, Jackson D, Jones L-M, Kaerst U, Kreft J, Kuhn M, Kunst F, Kurapkat G, Madueño E, Maitournam A, Vicente JM, Ng E, Nedjari H, Nordsiek G, Novella S, de Pablos B, Pérez-Diaz J-C, Purcell R, Remmel B, Rose M, Schlueter T, Simoes N, Tierrez A, Vázquez-Boland J-A, Voss H, Wehland J, Cossart P. 2001. Comparative genomics of *Listeria* species. Science (1979) 294:849–852.

5.	Mandin P, Geissmann T, Cossart P, Repoila F, Vergassola M. 2007. Identification of new noncoding RNAs in *Listeria monocytogenes* and prediction of mRNA targets. Nucleic Acids Res 35:962–974.
